# Topical Delivery of Cell-Penetrating Peptide-Modified Human Growth Hormone for Enhanced Wound Healing

**DOI:** 10.3390/ph16030394

**Published:** 2023-03-05

**Authors:** Tru Van Nguyen, Kyung-Hwa Lee, Yongzhuo Huang, Meong Cheol Shin, Yoon Shin Park, Hangun Kim, Cheol Moon

**Affiliations:** 1 College of Pharmacy and Research Institute of Life and Pharmaceutical Sciences, Sunchon National University, Suncheon 57922, Republic of Korea; 2 Department of Pathology, Chonnam National University Hwasun Hospital and Medical School, BioMedical Sciences Graduate Program (BMSGP), Hwasun 58128, Republic of Korea; 3 Shanghai Institute of Materia Medica, Chinese Academy of Sciences, Shanghai 201203, China; 4 College of Pharmacy and Research Institute of Pharmaceutical Sciences, Gyeongsang National University, Jinju 52828, Republic of Korea; 5 Department of Biological Sciences and Biotechnology, School of Biological Sciences, College of Natural Sciences, Chungbuk National University, Cheongju 28644, Republic of Korea

**Keywords:** cell-penetrating peptide, TAT peptide, topical delivery, human growth hormone, wound healing

## Abstract

Protein drugs have been emerging as a class of promising therapeutics. However, their topical application has been limited by their high molecular weight and poor permeability to the cell membrane. In this study, we aimed to enhance human growth hormone (hGH) permeability for topical application by conjugation of TAT peptide, a cell-penetrating peptide, to hGH via crosslinker. After TAT was conjugated to hGH, TAT-hGH was purified by affinity chromatography. TAT-hGH significantly increased cell proliferation compared with the control. Interestingly, the effect of TAT-hGH was higher than hGH at the same concentration. Furthermore, the conjugation of TAT to hGH enhanced the permeability of TAT-hGH across the cell membrane without affecting its biological activity in vitro. In vivo, the topical application of TAT-hGH into scar tissue markedly accelerated wound healing. Histological results showed that TAT-hGH dramatically promoted the re-epithelialization of wounds in the initial stage. These results demonstrate TAT-hGH as a new therapeutic potential drug for wound healing treatment. This study also provides a new method for topical protein application via enhancement of their permeability.

## 1. Introduction

Wound healing is a multi-step biological process that involves inflammation, tissue growth, and remodeling [1]. Various cells and factors affect the wound healing process. Among them, growth factors play a crucial role in this process [2,3,4]. Under high growth factor concentration in wound areas, fibroblast and capillary buds migrate toward the center of the wound. Growth factors improve re-epithelialization, epidermal differentiation, cell migration, proliferation, inflammatory response, dermal closure, matrix distribution, and skin remodeling throughout the healing process [4,5,6].

Human growth hormone (hGH) is a hydrophilic anabolic protein with a molecular weight of 22 kDa composed of 191 amino acids. hGH has been used to treat growth disorders in children and adult growth hormone deficiency. hGH increases cell proliferation, mitosis, and differentiation [7,8]. The effect of hGH is produced via interaction with its receptors, which are distributed on cell surfaces such as the liver or fibroblasts. It also stimulates the production of insulin-like growth factor 1 (IGF-1), which can accelerate the cell proliferation of keratinocytes and fibroblasts [2,4,9,10]. However, there is a lack of studies to apply hGH to wound healing treatment due to its high molecular weight and low skin permeability, although some studies have been conducted to improve its skin permeability through the use of absorption enhancers, microneedle, ultrasound, and conjugation with a specific peptide that has permeability through the cell membrane [7,11,12,13,14]. Among them, cell-penetrating peptide-mediated delivery is of particular interest due to their unique ability to translocate across plasma membranes efficiently and carry a wide range of macromolecular cargoes with them.

Cell-penetrating peptides (CPPs), also known as protein transduction domains (PTDs), were first reported in 1988 by two independent research groups: Green and Loewenstein, as well as Frankel and Pabo [15,16]. They identified the trans-activator of transcription (Tat) protein composed of 86 amino acids encoded by the human immunodeficiency virus type 1 (HIV-1), which translocates cell membranes and gains entry into mammalian cells. In addition to the full-length Tat protein, short peptides spanning the Tat basic domain (TAT) fused to different cargoes have also been shown to enter cells [17,18]. Tat is an intrinsically disordered protein that plays a pivotal role in regulating viral transcription. Its basic domain (49–57 aa, RKKRRQRRR), which contains positively charged arginine and lysine residues, is highly conserved and serves as an RNA binding motif, nuclear localization signal, and protein transduction domain [19]. Over the years, additional CPPs have been identified, such as Antp, VP22, and transportan [20]. These peptides typically comprise fewer than 30 amino acids and can facilitate cellular entry. To date, nearly 2000 CPPs have been identified using in silico prediction algorithms, which have facilitated peptide screening [21,22].

Various types of cargo, including liposomes, polymeric nanoparticles, lipid-based nanocarriers, dendrimers, and biomacromolecules, can be delivered into cells using CPPs [20,23,24]. CPPs can be categorized based on the type and arrangement of amino acids, such as cationic, amphipathic, and hydrophobic peptides, as well as their origin [25]. Various mechanisms have been proposed to explain how CPPs enter cells, but the exact mechanism is not yet fully understood [26]. The efficiency of CPP uptake by cells depends on several factors, including the type of cell, the specific CPP used, the cargo being delivered, and the concentration of the CPP [25]. Cationic CPPs such as TAT are composed of positively charged amino acids, including arginine, lysine, and histidine. These peptides interact with negatively charged motifs, such as heparan sulfate glycosaminoglycan and phospholipids on the plasma membrane, through a receptor-independent mechanism [26]. The concentration of CPP is also essential, as lower concentrations tend to promote endocytosis, while higher concentrations may result in direct penetration into cells, particularly for primary hydrophobic peptides and many cationic CPPs [27,28]. Despite the effectiveness of CPPs as vectors for intracellular delivery of macromolecules, their nonspecific targeting limits their systemic application, requiring more elaborate delivery systems for clinical use [26]. However, the topical application of CPPs holds potential as an effective tool for the local delivery of macromolecules. Topical delivery of genetically CPP-fused proteins, such as Rac1, a small GTPase, acidic fibroblast growth factor (aFGF), epidermal growth factor (EGF), platelet-derived growth factor-A (PDGF-A), and IGF-1 has been investigated [29,30,31,32].

In this study, we investigated an effective approach to enhance the permeability of hGH to skin wounds. This was achieved by conjugating hGH with the TAT peptide (TAT-hGH) through a disulfide bond. The effect of TAT-hGH on fibroblast proliferation was evaluated in vitro using a trans-well culture system, where the upper well was fully populated with keratinocyte cell lines, while the lower well was plated with fibroblast cells. The in vivo wound healing effect of TAT-hGH was also evaluated in a skin regeneration model.

## 2. Results

### 2.1. Conjugation of TAT to hGH and Purification of TAT-hGH

**Figure 1:**
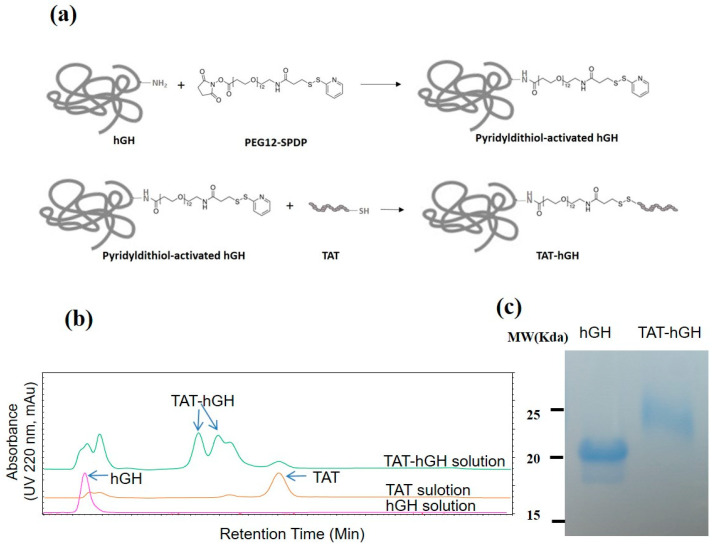
Conjugation of TAT to human growth hormone (hGH) using PEG12-SPDP crosslinker: (**a**) schematic illustration of the TAT-hGH conjugation step; (**b**) chromatograms of TAT-hGH purification using a heparin column; (**c**) 15% SDS-PAGE analysis of native hGH and purified TAT-conjugated hGH.

The scheme of the conjugation of TAT and hGH is shown in Figure 1a. To conjugate the TAT domain to the target protein hGH, we utilized a PEGylated, long-chain SPDP (PEG12-SPDP) crosslinker. By adopting this crosslinker, the hGH could be conjugated through its amine group to the sulfhydryl group of the cysteinyl TAT. Due to the TAT peptide’s abundant positively charged amino acids (lysine and arginine residues), the conjugate of hGH with TAT (TAT-hGH) could be effectively separated from the nonconjugated TAT and hGH using heparin affinity chromatography. The fraction containing the TAT-hGH conjugate was collected based on the appropriate peaks in the chromatogram with a distinct retention time. The typical HPLC chromatogram showed that TAT was successfully conjugated to hGH, as evidenced by the distinct peaks for TAT-hGH compared to either hGH or TAT alone (Figure 1b). The TAT-hGH peak was well separable from those of either TAT or hGH. As shown in Figure 1c, the purified TAT-hGH conjugate was also detected as a monomer with the expected size in the SDS-PAGE gel, distinct from hGH alone.

### 2.2. Skin Cell Proliferation Effect of TAT-hGH Conjugate

**Figure 2:**
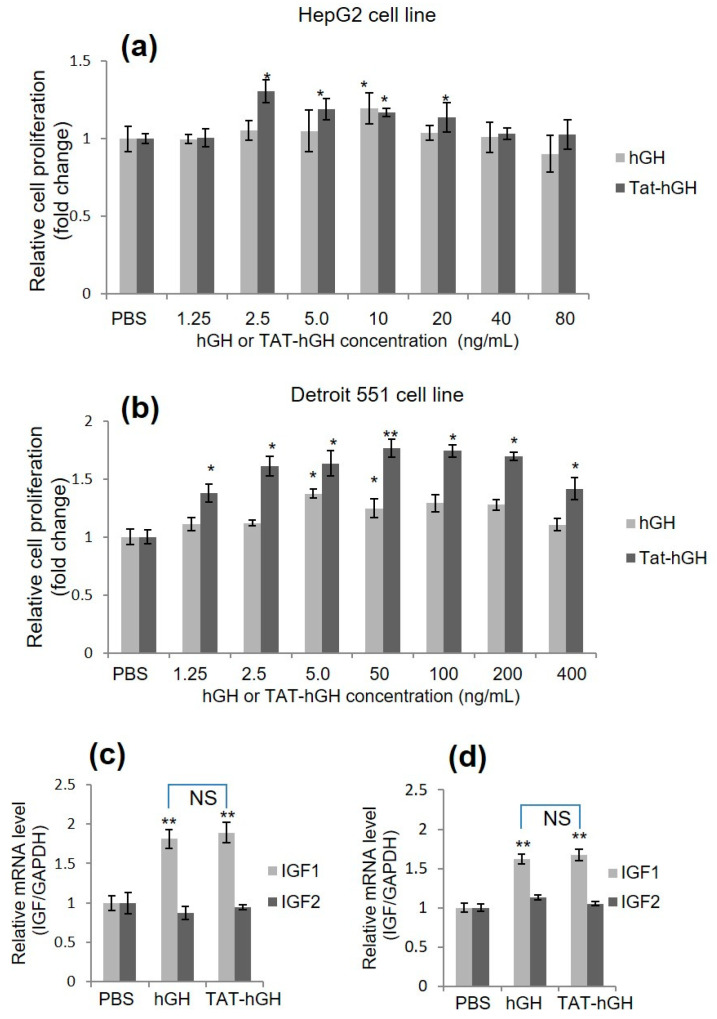
Relative cell proliferative activity and relative insulin-like growth factor (IGF) mRNA expression of human growth hormone (hGH) or TAT-conjugated hGH (TAT-hGH) in HepG2 and Detroit 551 cell lines over control. Cells were treated with hGH or TAT-hGH, and the relative cell proliferation and IGF mRNA expression were measured and normalized to control. (**a**) Proliferative activity in HepG2 cell line. (**b**) Proliferative activity in Detroit 551 cell line. (**c**) Relative IGF expression following treatment with hGH or TAT-hGH in HepG2 cell line. (**d**) Relative IGF expression following treatment with hGH or TAT-hGH in Detroit 551 cell line. Results are presented as the mean ± standard deviation (*n* = 3). NS indicates no significance; * indicates *p* < 0.05 versus the control (vehicle); ** indicates *p* < 0.01 versus the control (vehicle).

In order to evaluate the biological activity of TAT-hGH, we conducted a cell proliferation assay using two cell lines: HepG2 (a liver cancer cell, a major target organ for hGH activity) and Detroit 551 (a skin fibroblast cell line, a target cell type for topical application). Cells were treated with various concentrations of hGH or TAT-hGH and cell proliferation was measured using the MTT assay. Results showed that both hGH and TAT-hGH significantly increased cell proliferation (Figure 2a,b). Optimal concentrations of TAT-hGH were 2.5 ng/mL for HepG2 and 50 ng/mL for Detroit 551 cells, resulting in 1.30 and 1.76 times higher proliferation rates compared to the control vehicle, respectively.

Notably, TAT-hGH treatment showed significantly higher effects than hGH alone at the same concentration, particularly in Detroit 551 cells. These results indicate that the conjugation of TAT to hGH does not interfere with hGH’s biological activity and, in certain cell lines, enhances the ability for cell proliferation compared to hGH treatment alone (Figure 2b). This higher activity of TAT-hGH might be explained by the greater number of hGH binding to their cell surface receptors via TAT-mediated enhanced cell association. This is because the cationic nature of the TAT could mediate strong interaction with the negatively charged cell membrane via glycosaminoglycans or phospholipids.

### 2.3. Effect of TAT-hGH Conjugate on IGF mRNA Level

To demonstrate that hGH enhances cell proliferation, we evaluated growth-signaling activity using various methods. Similar to other growth-signaling processes, hGH binds directly to its receptor on target cells and triggers signals via a series of phosphorylation events to multiple downstream targets. The insulin-like growth factor (IGF) gene is known as one of the downstream factors in the hGH signal transduction pathway. We first assessed whether the expression of the IGF gene was regulated by the TAT-hGH conjugate in a manner similar to hGH. Upon treatment with TAT-hGH or hGH, both HepG2 and Detroit 551 cells showed significantly increased IGF-1 mRNA levels, while IGF-2 mRNA levels remained unchanged compared to the control group (Figure 2c,d). Furthermore, treatment with TAT-hGH or hGH alone did not result in significant differences in IGF-1 mRNA levels, indicating that the TAT-hGH conjugate has comparable biological activity to the same amount of hGH.

### 2.4. In Vitro Skin Permeability Study of TAT-hGH

**Figure 3:**
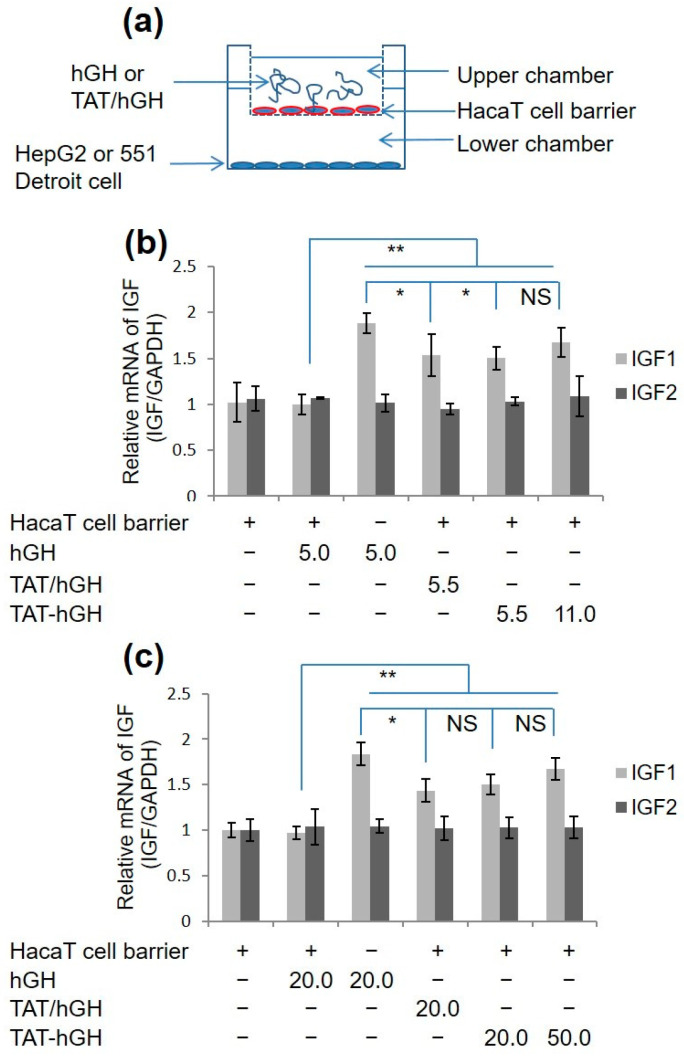
IGF mRNA expression in fibroblast cell lines treated with hGH, a mixture of TAT and hGH (TAT/hGH), and TAT-hGH. (**a**) Schematic illustration of the cell membrane permeability model using a trans-well system. (**b**) IGF mRNA expression in the HepG2 cell line. (**c**) IGF mRNA expression in the 551 Detroit cell line. Values are presented as the mean ± standard deviation (*n* = 3). NS indicates no significance; * indicates *p* < 0.05 vs. the control (vehicle); ** indicates *p* < 0.01 vs. the control (vehicle).

To evaluate the skin permeability of TAT-hGH, a cell barrier was generated by plating the HaCaT cell line on the top well of the trans-well culture system, which prevented TAT-hGH from directly contacting the target cells, HepG2 or Detroit 551 (Figure 3a). When hGH or TAT-hGH was treated with the HaCaT cell barrier in the top chamber, the IGF-1 mRNA levels extracted from HepG2 or Detroit551 cells in the bottom chamber were significantly increased compared to the PBS-treated cells (Figure 3b,c). However, there was no significant difference in IGF-1 mRNA levels between the PBS-control and hGH alone groups when both were plated with the HaCaT cell barrier on the top chamber (Figure 3b,c).

These results indicate that hGH alone cannot directly cross the cell membrane. Instead, TAT-hGH effectively penetrates the HaCaT cell barrier, increasing the IGF-1 mRNA levels of the target cells in the bottom chamber by up to 1.6 times higher than the negative control group. This demonstrates that the TAT domain allows TAT-hGH to penetrate the cell layers without compromising its biological activity.

### 2.5. In Vivo Wound Healing Study with TAT-hGH

The effect of TAT-hGH on wound healing was evaluated in vivo using C57BL/6 mice. A 4 mm biopsy punch was used to generate wound areas, and the degree of healing was observed daily after topical treatment with hGH, a mixture of TAT and hGH (TAT/hGH), and TAT-hGH. A hypromellose gel formula containing either hGH, TAT/hGH, or TAT-hGH at 100 ng/mL was applied to the wound area, respectively.

**Figure 4:**
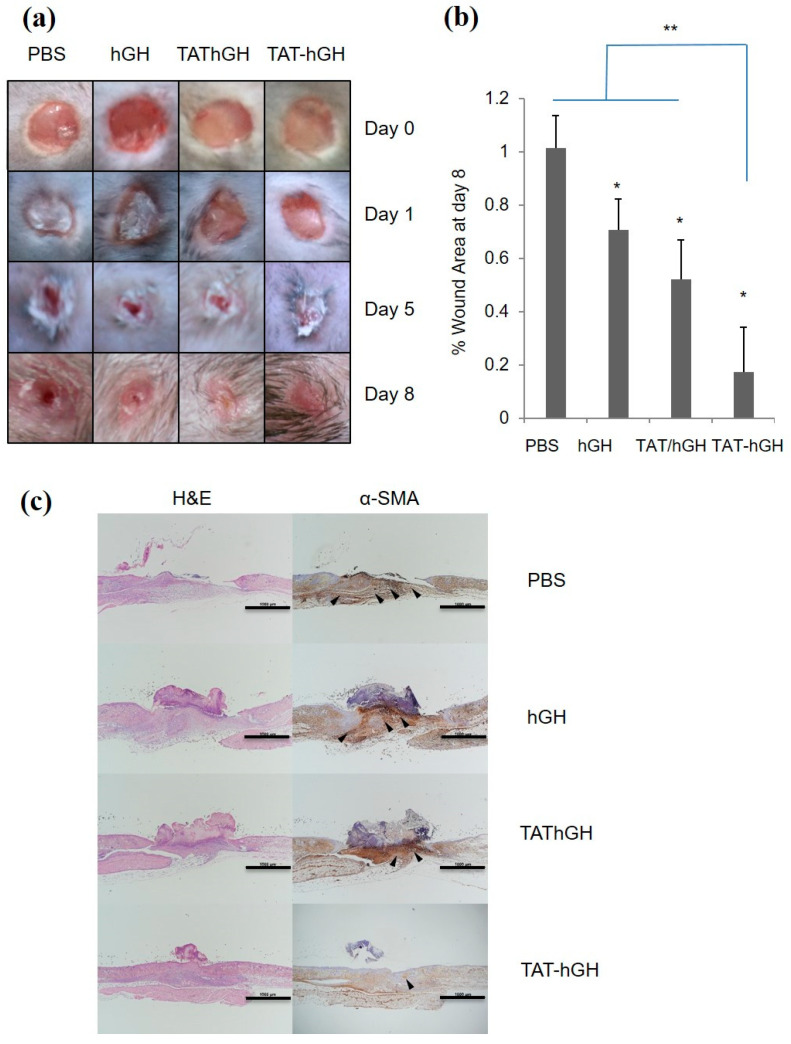
The effects of topical hypromellose gel treatments containing hGH, TAT/hGH, and TAT-hGH on wound re-epithelialization and tissue formation in C57BL/6 mice (*n* = 4). (**a**) The morphological changes in wound area over 8 days. The initial wound diameter was 4 mm; (**b**) the percentage of wound area at day 8; (**c**) representative light microscopy images of sections from the center of the wound tissue at day 8. The wound sections were stained using hematoxylin and eosin (H&E) to observe re-epithelialization, and immunohistochemical staining of α-smooth muscle actin (α-SMA, arrowheads) to evaluate myofibroblast differentiation. Scale bars are 1000 µm. Values are presented as the mean ± standard deviation (*n* = 4). * indicates *p* < 0.05 vs. the control (vehicle); ** indicates *p* < 0.01 vs. the control (vehicle).

As shown in Figure 4a, TAT-hGH treatment significantly accelerated wound healing compared to the vehicle-treated control group. The most significant difference among treatments was observed on the fifth day of treatment. On this day, the remaining wound sizes for TAT-hGH and hGH treatment were 37 ± 5% and 45 ± 4%, respectively, compared to 50 ± 7% for the control group. The wound size had completely recovered by the eighth day in the TAT-hGH treatment group, whereas it remained at 10% in the control group (Figure 4a,b). Furthermore, the histological analysis showed that the TAT-hGH gel formula significantly increased re-epithelialization in the early stages of treatment. On day 5, re-epithelialization had completely recovered over the wound area in the TAT-hGH-treated group, while only 30% epidermal recovery was found in the control group. On day 8, enhanced regeneration of the wound skin after TAT-hGH treatment was observed microscopically through hematoxylin and eosin (H&E) staining (Figure 4c). The wound tissue with PBS treatment left an area in denuded status without complete re-epithelialization in the center of the lesion. On the other hand, the epithelial layer was almost restored in hGH or TAT/hGH samples, and the skin structure on top of epithelialization was recovered the most in the TAT-hGH sample.

Moreover, the immunostaining analysis of α-smooth muscle actin (α-SMA) in the wound area indicated an increase in activated fibroblasts (myofibroblasts), which are markers for wound closure and scar formation. Excessive myofibroblast proliferation plays a role in inhibiting re-epithelialization through scar formation. The α-SMA-positive myofibroblast population was most prominent in the PBS sample, became relatively small in hGH or TAT/hGH, and was very rare in TAT-hGH specimens. Taken together, the results demonstrate that skin regeneration was almost completed by TAT-hGH compared to TAT/hGH mixture, hGH, or the control.

## 3. Discussion

During wound healing, various biological macromolecules such as growth factors and extracellular matrix (ECM) proteins play critical roles in skin tissue regeneration. Growth factors such as growth hormone-releasing hormone (GHRH), epidermal growth factor (EGF), and human growth hormone (hGH) stimulate cell proliferation and differentiation at wound sites, leading to tissue restoration [29,30,33]. However, applying these growth factors directly to wounds is limited by poor skin permeability and short in vivo half-life. To overcome these challenges, various methods have been tried to increase their skin permeability, such as microneedle patches, iontophoresis, chemical enhancers, ultrasound, and cell-penetrating peptides (CPPs).

Among these, the use of CPPs has received considerable attention due to their benefits, such as low irritation and minimal disruption of the *stratum corneum* lipid structure, effectively enhancing macromolecule skin permeability. CPPs are also easy to apply, as they do not require special equipment or techniques [34]. In addition, CPPs can effectively deliver various high-molecular-weight cargoes into cells, regardless of the target cell type [35,36]. In this study, the TAT peptide was conjugated to hGH using chemical conjugation instead of constructing a fusion protein. This approach ensured that TAT did not interfere with hGH’s biological activity, as seen from the results of cell proliferation and IGF-1 expression [37].

hGH stimulates the production and release of IGF-1 from various tissues, primarily the liver [7]. IGF-1 is also expressed in skin wounds by keratinocytes, fibroblasts, and macrophages and plays a critical role in epidermal and dermal wound healing by promoting the proliferation and migration of keratinocytes, endothelial cells, and fibroblasts [38]. However, it has been reported that at extremely high concentrations (500 ng/mL) of hGH, IGF-1 mRNA expression is down-regulated [7]. To optimize the concentration for our study, we selected 2.5 ng/mL of TAT-hGH for HepG2 cells and 50 ng/mL for Detroit 551 cells. Consistent with previous reports on TAT-mediated enhanced skin penetration effect, our study on the permeability of HaCaT cell barriers revealed significantly higher mRNA expression of IGF-1 in TAT-hGH compared to hGH alone, indicating that TAT improves keratinocyte permeability without compromising biological activity [31,32].

The penetration mechanisms of CPPs are not yet fully understood. TAT’s high positive-charge content from amino acids such as lysine and arginine seems to allow it to interact efficiently with the negatively charged glycosaminoglycan or lipids on cell membranes [26,27,39]. Hydrophobic interactions between TAT’s hydrophobic residues and the plasma membrane surface also play a role. These interactions increase the concentration of TAT-hGH on the cell surface and enhance skin permeability. Another possible mechanism for transcellular delivery of TAT peptides includes guanidine head-mediated hydrogen bonding formation between arginine and the lipid phosphate on the membrane, as well as the interaction of arginine with extracellular matrices, which could lead to the disruption of the ordered lipid orientation and create a concentration gradient through open channels [30].

Maintaining high hGH concentrations in the wound area is crucial for effective wound healing. In this study, TAT-hGH was formulated into a gel using hypromellose to extend its retention and stability on the skin. The results showed that TAT-hGH gel was more effective than TAT/hGH mixture in promoting fibroblast proliferation and keratinocyte proliferation as indicated by increased IGF-1 gene expression. To further improve the stability of the TAT-hGH or enhance the residence time on the wound area, adopting a nanoparticle-based delivery system or an adhesive-type formulation might be effective [40,41].

The skin undergoes a process of re-epithelialization during wound healing. It is important that the transition of fibroblasts to myofibroblasts is reduced to promote proper healing and minimize scarring. While myofibroblasts play a crucial role in tissue repair, excessive activation can impede the healing process and lead to increased scarring. By reducing the number of activated myofibroblasts, the skin can better heal without excessive scarring [42,43]. Alpha-smooth muscle actin (α-SMA) is a commonly used marker for myofibroblast activation and its expression indicates the transition of fibroblasts to myofibroblasts [43,44]. A decrease in α-SMA expression during wound healing usually signifies a reduction in myofibroblast activation and is a sign of proper healing with reduced scarring. Thus, the reduction of α-SMA expression by TAT-hGH may enhance wound healing by reducing scar formation.

## 4. Materials and Methods

### 4.1. Reagents

Human growth hormone (hGH), TAT peptide (CGGGYGRKKRRQRRR), and pegylated long-chain succinimidyl 3-(2-pyridyldithio)propionate crosslinker (PEG12-SPDP) were obtained from LG Life Science Ltd. (Seoul, Republic of Korea), Shanghai Leon Chemical Ltd. (Shanghai, China), and Thermo Fisher Scientific (Waltham, MA, USA), respectively. Hypromellose (Metolose^®^ 90SH-4000, 4000 mPa.s, Shin-Etsu Chemical Ltd., Tokyo, Japan) was kindly donated by Richwood Trading Co., Ltd. (Seoul, Korea), and Thiazolyl Blue Tetrazolium Bromide (MTT) and dimethyl sulfoxide (DMSO) were obtained from Sigma-Aldrich (St. Louis, MO, USA). Dulbecco’s modified essential medium (DMEM), fetal bovine serum (FBS), 1% penicillin and streptomycin (Pen/Strep), and Eagle’s minimum essential medium (EMEM) were purchased from Gen Depot (Katy, TX, USA). All reagents used were of analytical grade.

### 4.2. Cell Culture

Hep G2 (human hepatoma) and HaCaT (immortalized human keratinocyte) cell lines were maintained in DMEM supplemented with 10% FBS and 1% penicillin and streptomycin. Detroit 551 cell line (skin fibroblast cell line) was maintained in EMEM supplemented with 10% FBS and 1% penicillin and streptomycin. Cells were cultured in a humidified atmosphere containing 5% CO_2_ at 37 °C. The Hep G2 and Detroit 551 cell lines were sourced from the Korean Cell Line Bank (Seoul, Republic of Korea) in the Republic of Korea, while the HaCaT cell line was obtained from the American Type Culture Collection (Manassas, VA, USA).

### 4.3. Preparation and Purification of TAT-hGH

PEG12-SPDP, a crosslinker for the conjugation of TAT to hGH, was dissolved in DMSO. PEG12-SPDP solution was slowly added to hGH in PBS in a 5:1 molar ratio and incubated for 2 h at room temperature with gentle mixing. Excess nonreacted PEG12-SPDP reagent was removed by ultrafiltration (MWCO 10,000). PBS-diluted TAT was then added to the mixture in a 10:1 molar ratio with PEG-SPDP-hGH and incubated overnight at room temperature. The conjugate of hGH with TAT (TAT-hGH) was then purified by affinity chromatography (Agilent 1260, Agilent Technologies, Santa Clara, CA, USA) with a heparin column (GE Healthcare Bio-Sciences AB, Uppsala, Sweden). The mixture was eluted with a two buffer system with the flow rate of 1 mL/mL. Solvent A was 10 mM sodium phosphate buffer pH 7.2, and solvent B was 2 M NaCl with 10 mM sodium phosphate buffer pH 7.2. The gradient started with 0% B and raised to 60% B within 10 min, then to 100% B within 15 min, followed by 3 min at 100% A. TAT-hGH peak was detected with a UV detector the wavelength of which was 220 nm. The purification of TAT-hGH was confirmed by SDS-PAGE staining with Coomassie Brilliant Blue R250.

### 4.4. Biological Activity of TAT-hGH

The biological activity of TAT-hGH was tested using a cell proliferation assay on HepG2 and 551 Detroit cell lines. HepG2 and 551 Detroit cell lines were seeded at a density of 5 × 10^3^ cells per well in 135 µL of DMEM or EMEM medium, respectively, containing 10% FBS and 1% penicillin/streptomycin in a 96-well plate. The cells were incubated at 37 °C for 12 h to allow for attachment to the well. The cells were then treated with various concentrations of hGH or TAT-hGH, while PBS served as a control. After 24 h of incubation, the rate of cell proliferation was analyzed using the MTT assay. Fifteen microliters of MTT solution (0.5 mg/mL) were added to each well and incubated for an additional 4 h at 37 °C. The MTT solution was removed, and 150 µL of DMSO was added to each well to dissolve the formed crystals. The optical density was measured using an automated microplate reader at 570 nm wavelength.

### 4.5. Quantitative RT-PCR

The quantitative RT-PCR (qRT-PCR) was performed as previously described. Briefly, HepG2 and Detroit 551 cells were cultured to the density of 1 × 10^5^ cells/mL. After 12 h, the cells were washed three times with PBS and treated with various concentrations of hGH or TAT-hGH in DMEM or EMEM medium without FBS. After 18 h, total RNA was isolated using RNAiso Plus (TaKaRa, Otsu, Shiga, Japan) according to the manufacturer’s instructions. Total RNA from each group of treated cells was converted to cDNA using an M-MLV reverse Transcriptase kit (Invitrogen, Carlsbad, CA, USA) and SYBR green (Enzynomics, Seoul, Korea). The primers used for real-time PCR were IGF-1 (forward) 5′-TGGATGCTCTTCAGTTCGTG-3′ and (reverse) 5′-TGGTAGGGG-GCTGATAC-3′; IGF-2 (forward) 5′-ACACCCTCCAGTTCGTCTGT-3′, and (reverse) 5′-GGGGTATCTTGGGGAAGTTGT-3′. qRT-PCR reactions and analyses were performed using CFX (Bio-Rad, Hercules, CA, USA).

### 4.6. In Vitro Cell Permeability Study

A co-culture model was constructed using a trans-well chamber assay with a top chamber made of an 8 µm pore-size polycarbonate membrane coated with 1% gelatin (Corning Costar, Corning, NY, USA). The HepG2 and Detroit 551 cell lines were plated at 1 × 10^5^ cells/well in a culture medium with 10% FBS and 1% penicillin and streptomycin in the lower compartment. The upper compartment was filled with 300 µL of culture medium containing HaCaT cells (2.38 × 10^6^ cells/mL) to form a barrier and prevent direct contact between hGH or TAT-hGH and the HepG2 and Detroit 551 cell lines. The cells were incubated for attachment and stability for 12 h, after which the media in both compartments was replaced with fresh medium without FBS. hGH or TAT-hGH of various concentrations were added to the upper compartment. The cells were incubated for an additional 18 h. The cells from the lower compartment were harvested, and total RNA was extracted using 100 µL of TRIzol reagent according to the manufacturer’s protocol. The cells in the upper compartment were fixed and stained with the Diff Quick kit (Sysmex, Kobe, Japan) to confirm complete coverage of the HaCaT cell line.

### 4.7. TAT-hGH Gel Formulation for Topical Application

TAT-hGH gel formulation was made for topical treatment by using hypromellose. The final concentration of hypromellose was 0.9% after it was added to hGH, a physical mixture of TAT/hGH, and a TAT-hGH solution in PBS (100 ng/mL). The mixture solutions were gently stirred using a waving shaker (Jeio Tech, Daejeon, Korea) at 8 rpm at 40 °C for 12 h. A PBS solution served as a control vehicle.

### 4.8. In Vivo Wound Healing Analysis

The in vivo wound healing assay was conducted as previously described [4]. Six-week-old C57BL/6 male mice were purchased from Orient Bio (Seongnam, Korea) and were housed in standard polycarbonate cages under controlled conditions at 22 ± 2 °C and 50 ± 5% humidity, with a 12 h light/dark cycle. Each mouse was housed separately and provided with commercial rodent chow (DAE-HAN Biolink) and water during the experimental period. Prior to the experiments, the mice were allowed to acclimate to the laboratory conditions for 1 week. To create wounds, the mice were anesthetized with 100 mg/kg ketamine and 10 mg/kg xylazine and a 4 mm biopsy punch was used to create 4 wounds on their backs, 2 on each side of the dorsal midline, at equal distances, to ensure equal tension over the mouse back and avoid influencing the wound contraction. The wounds were treated twice daily for 8 days with 20 μL of the formulated TAT-hGH (100 ng/mL), a mixture of TAT and hGH (100 ng/mL), and hGH (100 ng/mL) vehicles. A PBS-formulated gel served as a control vehicle. The exact volume of gel formulations was obtained using a micropipette and was applied directly to the wound areas, left uncovered.

Wound sizes were measured before applying the samples using electronic vernier calipers (Mitutoyo, Kawasaki, Japan), and pictures were taken at day 0, 1, 5, and 8 after treatment using a digital camera (Canon, Tokyo, Japan) to visualize the changes in the wound size. The degree of wound healing was expressed as the percentage of the remaining wound area. The excised wound tissue was fixed in 10% neutral-buffered formalin and then dissected and embedded in paraffin. Tissue blocks were sectioned at 3 μm thickness using a microtome and stained with hematoxylin and eosin (H&E). Unstained slides from each tissue block were immunostained with specific antibodies against α-smooth muscle actin (α-SMA, dilution 1:100, clone HHF35, DakoCytomation, Glostrup, Denmark) using a Bond-Max Autostainer (Leica Microsystems, Buffalo Grove, IL, USA). The slides were evaluated by an experienced pathologist (KHL) and photographed using the Nikon DS-Ri2 system (Nikon Japan, Tokyo, Japan).

### 4.9. Statistical Analysis

The data are presented as mean and standard deviation, and the Student’s *t*-test was used to compare the means between different groups. A *p*-value of less than 0.05 was considered statistically significant.

## 5. Conclusions

In conclusion, the results of this study demonstrate that the TAT conjugation of hGH enhances the permeability of hGH to cell membranes without affecting its biological activity in vitro. In vivo, topical application of TAT-hGH to scar tissue significantly accelerated wound healing, as shown by the histological results. The results indicate that TAT-hGH dramatically promoted re-epithelialization of wounds during the initial stage of wound healing. However, further research is needed to fully understand the mechanisms of TAT-hGH penetration and its long-term effects on skin health and wound healing. These findings have implications for developing more effective treatments for skin wounds and scarring.

## Data Availability

Data is contained within the article.
